# The War and Dietaries in Poor-Law Institutions

**Published:** 1915-07-10

**Authors:** 


### THE WAR AND DIETARIES IN POOR-LAW INSTITUTIONS.

### The Proper Way to Economise.

The need for economising our resources is now generally recognised. Naturally, the matter has been the subject of consideration by many Boards of Guardians. Judging from the discussions which have been reported, the method of economy most frequently adopted has been an alteration in the dietary. To this there can be no reasonable objection, provided the changes suggested have been carefully thought out with a full knowledge of all the factors which enter into a wise solution of the problem. The conservation of our food supplies and the avoidance of all unnecessary waste in their use are undoubtedly of the greatest importance to the nation. Unfortunately, most of the discussions have turned not so much upon the possible means of preventing waste as upon the substitution for meat of cheaper and less palatable substances.

### Science and Economy.

The matter is one which concerns the medical officer of a Poor-Law institution very closely. The dieting of the inmates of the sick wards is entirely in his hands, and in the case of the other inmates the Guardians must obtain his advice in writing before varying the diet. The Guardians' powers are further limited by the fact that they must keep within the general regulations laid down by the Local Government Board in the Poor-Law Institutions Order. It behoves, therefore, the medical officer very carefully to consider any alterations proposed and to refuse his sanction to any suggestions likely to be detrimental to the well-being of the inmates, whilst directing the Guardians along lines which are really economical and scientifically sound. Most careful consideration is required, because the dietaries of Poor-Law institutions are not luxurious, and in the case of the workhouse inmates have been framed so as to approximate to the minimum required for the preservation of the health of an average man or woman at work.

### Some New War Menus.

The changes which have commended themselves to most Boards of Guardians are similar to those adopted by the Halifax Guardians, described in our issue of June 19. They consist of the substitution of bread, cheese, and coffee for one of the meat dinners once a week, and of pea-soup and bread for Irish stew on another day. It is impossible to say in this particular instance whether these changes are free from objection, because the whole of the dietary for the week and the ingredients for the pea-soup are not given, and there is no indication of the amount of saving the changes will effect. It should be noted, however, that in the Billericay Union a proposal to substitute bread and cheese and coffee for meat was rejected, because the saving at present prices was calculated to amount to only six shillings a week. As regards dietetic value no objection can be raised to the use of bread and cheese in place of meat. The sole objection to it, and an important one, is the relative palatability of the two meals. Bread and cheese and coffee—especially coffee as usually made in England—do not form an appetising combination. To make the meal palatable the bread and cheese must be of the best, butter should be added, and the drink should be beer. Then one has a meal of high nutritious value and one no *gourmet* despises, but the meal is not a cheap one.

### The Practical Remedy.

As regards the majority of our workhouse dietaries, indeed, economy is to be sought not so much in a change of ingredients as in a change in the methods of cooking and serving and in the prevention of waste. More use should be made of boiled meats and of stews. Roasting and baking involve a great deal of waste, which is avoided when the meat is boiled or stewed. Fish, when prices are not abnormally high as they are just now, forms a nutritious, a palatable, and an economical substitute for meat. Vegetables should be more largely used, and care should be taken to avoid waste in trimming them. In the case of potatoes, the wasteful custom of peeling them before boiling should be discarded. In some parts of Europe pea-shells are stewed with meat and potatoes, and the result is a perfectly palatable and nutritious dish. It is perhaps too much to expect the English cook to do this, but it is an illustration of the amount of valuable food we habitually waste. One may, however, with more hope of success, propose the greater use of soups and advocate the introduction of a stock-pot into every institution. Bones, the outer leaves of vegetables, pieces of bread, and much other material which usually finds its way to the pig-bucket can, and should be, utilised as the basis of nutritious and palatable soups and stews. Lastly, the Local Government Board regulations requiring the weighing of each portion served to an inmate should be cancelled. This has already been done in the case of bread, and a considerable saving has been effected thereby. A similar saving would occur if each inmate was served according to appetite. Under the present system many inmates are served with quantities they do not need, whilst others receive less than their wants.

